# *Schistosoma japonicum* Egg Specific Protein SjE16.7 Recruits Neutrophils and Induces Inflammatory Hepatic Granuloma Initiation

**DOI:** 10.1371/journal.pntd.0002703

**Published:** 2014-02-13

**Authors:** Chenyun Wu, Qing Chen, Yan Fang, Jianhua Wu, Yanyan Han, Ying Wang, Yang Yang, Min Chu, Yan Feng, Linping Tan, Xiaokui Guo, Wei Hu, Zhaojun Wang

**Affiliations:** 1 Department of Microbiology and Parasitology, Shanghai Jiao Tong University School of Medicine, Shanghai, China; 2 Institute of Health Sciences, Shanghai Institutes for Biological Sciences of Chinese Academy of Sciences/Shanghai Jiao Tong University School of Medicine, Shanghai, China; 3 The Ninth People's Hospital Affiliated to Shanghai Jiao Tong University School of Medicine, Shanghai, China; 4 Department of Microbiology and Microbial Engineering, School of Life Science, Fudan University, Shanghai, China; Federal University of Minas Gerais, Brazil

## Abstract

Neutrophils are known to play a major role in the egg granulomatous lesions caused by *Schistosoma japonicum*, but the precise mechanism by which eggs recruit or active neutrophil is unknown. Here we report *S. japonicum* egg specific EF-hand protein-SjE16.7 is a potent neutrophil recruiter and initiates the egg associated inflammatory granuloma in schistosomiasis. We show that the expression of SjE16.7 at level of both mRNA and protein is restricted to the egg stage. It locates in the miracidium and subshell area of the egg and can be secreted by the egg. The antigenic properties of SjE16.7 strongly suggest a role for SjE16.7 as an egg-derived molecule involved in host-parasite interactions. To study SjE16.7 functions *in vivo*, we challenged murine air pouch with recombinant SjE16.7. The results showed SjE16.7 trigged more inflammatory cell infiltration than vehicle or control protein. Using peritoneal exudate neutrophils from mice, we found that SjE16.7 significantly induced neutrophil chemotaxis *in vitro*, and the observed phenotypes were associated with enhanced Rac GTPase activation in SjE16.7 treated cells. Finally, *in vivo* hepatic granuloma formation model showed SjE16.7 coupled beads recruited more inflammatory cell infiltration than control beads. Our findings suggest SjE16.7 is an important pathogenic factor derived from egg. By recruiting neutrophils and inducing local inflammation, SjE16.7 facilitates eggs to be excreted through gut tissues and also initiates pathology in the liver; therefore SjE16.7 is a possible target for the prevention and treatment of schistosomiasis.

## Author Summary

As a neglected disease, schistosomiasis continues to be a significant cause of parasitic morbidity and mortality worldwide. *Schistosoma japonicum* is one of the major causative agents of human schistosomiasis. Trapped in the liver or intestinal tissue, *S. japonicum* eggs are the main cause of pathology following infection. They induce vigorous immune responses from the host, which facilitate the passage of the eggs from the tissue to the gut lumen and cause the pathology in liver. In this paper, we described, for the first time, *S. japonicum* egg specific EF-hand protein-SjE16.7 is a potent neutrophil recruiter and initiates the egg associated inflammatory granuloma in schistosomiasis. This study presents a precise mechanism by which eggs recruit neutrophil and induce inflammatory response. It furthers our understanding of the immunopathogenesis of human schistosomiasis. In addition, it provides a potential target for the prevention and treatment of this globally important parasite.

## Introduction

Schistosomiasis is a neglected tropical disease caused by trematode parasites of the genus *Schistosoma*. It is estimated to affect 200 million people globally and cause nearly 280,000 deaths reported annually [1]. *S. japonicum* is the major causative agent of schistosomiasis in South East Asia and China, which mainly cause “intestinal” and “hepatic schistosomiasis”. Deposited in the host liver or intestinal tissue, schistosome eggs are the major cause of pathology in schistosomiasis. They are viable metabolically active organisms, and highly antigenic. Eggs evoke inflammation leading to a granulomatous response that is necessary for its translocation into the intestinal lumen and excretion in the feces. Meanwhile this process initiates the pathology in host liver and intestine [2].

Neutrophils are believed to play a significant role in *S. japonicum* granulomatous pathology [3]–[5]. In the initiation of granuloma formation, neutrophils are recruited to the core of granuloma leads a neutrophil-mediated inflammatory response, which ultimately cause tissue damage [4]. At the later stage, neutrophils are recruited again and accumulated at the periphery of the granuloma, where they release a number of granule proteins involved in collagen degradation and reabsorption. It is well known, granuloma formation is a T cell-mediated immune response. The T cell-mediated response, especially CD4^+^ T cell response has been reported to participate in neutrophil induction in schistosomiasis [5], [6]. However, in CD4^+^ T cell depleted mice, neutrophils still can be observed and become the dominated population in the cellular infiltrate around the egg [7]. These results suggest that neutrophils can be attracted by T cell independent way. Using crude extracts or antigen fractions from *S. japonicum* egg, Owhashi and Horri showed schistosome egg components have high neutrophil chemotactic activity [8], [9], but up to now the detail molecule and mechanism involved in chemoattractants for neutrophils are still not identified yet.

Previously transcriptomic analyses of *S. japonicum* showed egg 16 kD calcium-binding protein (SjE16.7) is specifically expressed in eggs, but the function of this protein and whether it plays roles in egg associated pathogenesis are unknown [10]. In this study, we cloned SjE16.7 from *S. japonicum* eggs, and then studied its function in host innate immune response. We showed as an egg-derived molecule, SjE16.7 promotes neutrophil chemotaxis through Rac GTPase pathway. It plays impotent roles in schistosome egg induced inflammatory granuloma; therefore SjE16.7 is a potential target for prevention and treatment of schistosomiasis.

## Methods

### Ethics statement

The conducts and procedures involving animal experiments were approved by the Animal Ethics Committee of Shanghai Jiao Tong University School of Medicine (project number 2012008) according to Regulations for the Administration of Affairs Concerning Experimental Animals (approved by the State Council of the People's Republic of China) and Guide for the Care and Use of Laboratory Animals (Department of Laboratory Science, Shanghai Jiao Tong University School of Medicine, laboratory animal usage license number SYXK 2008-0050, certificated by Shanghai Committee of Science and Technology).

### Reagents

Chemicals were purchased from Sigma-Aldrich Co. unless otherwise noted. Ca^2+^, Mg^2+^, and phenol red-free Hanks balanced salt solution (HBSS), phosphate-buffered saline (PBS; PH7.2) and Dulbecco's Modified Eagle Medium (DMEM) were obtained from Life Technologies. NSC23766 was purchased from Tocris Bioscience. Polymyxin B was ordered for Sigma-Aldrich.

### Animals

Male C57BL6/J (6–7 week) mice were purchased from Shanghai Laboratory Animal Center, Chinese Academy of Sciences. Mice were housed under specific pathogen-free conditions and fed autoclaved food and water as needed. Male New Zealand White rabbits (2.0–2.5 kg) were provided and housed in the Department of Laboratory Animal Science, Shanghai Jiao Tong University School of Medicine.

### Parasite infection and isolation

C57BL6/J mice were percutaneously infected with 30 *S. japonicum* cercariae (Chinese mainland strain, Anhui population, National Institute of Parasitic Diseases, Chinese Center for Disease Control and Prevention). Adult worms were collected from infected mice 6 weeks post infection. Mice were perfused with PBS to remove worms from mesenteric veins. The male and female adult parasites were separated and stored in liquid nitrogen until use. Eggs were harvested from mouse liver. Tissues were minced with scissors in ice-cold 1.2% NaCl and passed through a crude sieve. The filtrate was passed through a series of sieves with decreasing pore size and finally eggs were collected from top sieve (45 µm). To collect the mature eggs, eggs were purified using a Percoll gradient, and then washed and concentrated by centrifugation. Eggs were used for circumoval precipitin test or stored in liquid nitrogen immediately.

### Total RNA extraction and first strand cDNA synthesis

Total RNA was extracted from male, female or coupled adult worms using an RNAeasy Mini Kit (Qiagen) following the instructions of the manufacturer. The kit was also used to extract total RNA from eggs. First strand cDNA was synthesized using a Sensiscript RT Kit (Qiagen).

### Cloning of SjE16.7

SjE16.7 gene sequence (NCBI/GenBank AY816133) was obtained from NCBI/GenBank. A pair of primers 5′- ATGTCGGATGAAAACCGATGGATTGC-3′ and 5′-TTATTCATACGTTTGACGTACATAAGC-3′ was designed to amplify the ORF of cDNA using the first strand cDNA from females, males, coupled adults and eggs respectively, as templates. The PCR products were separated by running an agarose gel and a DNA band matching the designated size was cut and extracted using a Qiaquick Gel Extraction kit (Qiagen). The DNA was then ligated into a cloning pGEM-T vector (Promega, Madison, WI). Positive clones were selected and sequenced.

### Expression and purification of recombinant SjE16.7

To express the eukaryotic SjE16.7 in vitro, we designed a pair of primers: 5′-CGGGAATTCATGTCGGATGAAAACC-3′ and 5′- ATTGCGGCCGCTTAGTGGTGGTGGTGGTGGTGTTCATACGTTTG-3′ to subclone the SjE16.7 into the pPIC9K vector (Invitrogen) and transformed in *Pichia pastoris* strain GS115 (Invitrogen) according to the manufacturer's instructions. The generated protein was fused with His tag at the C terminal for affinity purification. The recombinant His-SjE16.7 protein was collected from the yeast culture supernatant and purified by Ni-NTA Superflow Cartridges according to the manufacturer's instructions (Qiagen). The molecular weight and purity of recombinant proteins were identified by SDS-PAGE.

Prokaryotic SjE16.7 protein was expressed in *E. coli*. A pair of primes: 5′-CGGGAATTCATGTCGGATGAAAACC-3′ and 5′-ATTGCGGCCGCTTATTCATACGTTTG-3′ was used to amplify the target gene and subcloned into the pGEX-4T-1 plasmid (GE Healthcare life Sciences). The target gene was fused in frame with the N terminal GST tag. The plasmid was transformed into *E. coli* BL21 cells for protein expression. Protein expression was initiated by IPTG and cells were harvested after 4 h culture. Bacteria were lysed and sonicated. The recombinant fusion protein GST-SjE16.7 or GST control protein from *E. coli* lysates was purified using Glutathione Sepharose 4B (GE Healthcare Life Sciences). LPS from prokaryotic expressed proteins was removed by Triton X-114 phase separation as literature described [11]. Briefly, Triton X-114 was added to the proteins to a final concentration of 1% and incubated for 30 min at 4°C with constant stirring, followed by 10 min incubation at 37°C and a centrifugation step at 16,000×g at 25°C, for 10 min. Six cycles of Triton X-114 phase separation was performed for a sufficient LPS depletion. Trace amounts of Triton X-114 were removed by dialysis against PBS. Finally LPS content was detected by Tachypleus Amebocyte Lysate Kits (Gulangyu, Xiamen China) according to the manufacturer's recommendation.

In some experiments, purified GST-SjE16.7 was cleaved with thrombin (GE Healthcare life Sciences) to remove the GST tag. GST free SjE16.7 protein was used in ELISA. GST-SjE16.7 fusion protein was used in other experiments (air pouch model, transwell migration assay, PBD pull-down assay and inflammatory hepatic granuloma model), while GST protein was used as control.

### Preparation of SjE16.7 antiserum and antibody

SjE16.7 antiserum was prepared in C57BL6/J mice and New Zealand White rabbits. Recombinant SjE16.7 was formulated with either Freunds complete (primary) or Freunds incomplete (two boosts at two weekly intervals) adjuvants and the preparations were subcutaneously injected into the animals. Animals were sacrificed 2 weeks after the final antigen immunization and sera were collected from the blood. Antibodies were purified from rabbit anti-SjE16.7 sera. Immunoglobulins were precipitated with ammonium sulfate first, and then purified using Protein A/G agarose beads according to the product's instruction (Pierce Biotechnology)

### Preparation of soluble egg antigen and adult worm antigen

Soluble egg antigen (SEA) and adult worm antigen (AwAj) of *S. japonicum* were prepared as described previously [12]. Briefly, eggs or adult worms were suspended in PBS and homogenized in an ice-chilled water bath. The mixture was centrifuged at 100,000 g for 1 h. The supernatant was used as SEA or AwAj. Protein concentration was measure by standard Bradford protein assay (Biorad) using bovine serum albumin as a standard.

### SDS-PAGE and western blot

SDS-PAGE was performed by the procedure of Laemmli using 10–12% polyacrylamide gels in presence of 5% 2-mercaptoethanol in sample buffer. Protein molecular-weight markers (Fermentas or Biorad) were used as MW standards. Proteins were visualized by Coomassie brilliant blue staining. Western blotting was performed on nitrocellulose filter (Biorad). Blots were immunostained with 100 times diluted mouse anti-SjE16.7 serum, and 1000 times diluted horseradish peroxidase (HRP) conjugated goat anti-mouse IgG (Cell Signaling). Enhanced chemiluminescence (ECL, Pierce) was used as substrates and signals were analyzed by Luminescent imager (ImageQuant Las 4000, GE Healthcare).

### Circumoval precipitin test (COPT)

*S. japonicum* eggs were isolated from the livers of infected mice. Live eggs were cultured at 37°C with normal rabbit sera, rabbit anti-SjE16.7 sera or sera from *S. japonicum* infected rabbits. Eggs were examined for the presence of precipitates around eggs 48 h later.

### Immunohistochemistry

Liver specimens were fixed in 10% formalin, embedded in paraffin and sectioned at 3 µm. Following antigen retrieval by boiling in 0.01 M sodium citrate, pH 6, for 20 minutes in water bath, endogenous peroxidase activity was blocked by incubation with 3% (v/v) H_2_O_2_ for 20 min at room temperature. Slides were washed three times with PBS, and blocked with 5% bovine serum albumin for 20 min at room temperature. Tissue sections were then probed with 1∶50 diluted rabbit anti-SjE16.7 sera for 1 hour at 37°C. Slides were washed three times with PBS, and HRP conjugated secondary antibody was added (anti-rabbit IgG in MaxVisionTM HRP-Polymer anti-Rabbit IHC Kit, Maxim, Fujian, China) and incubated for 12 min at room temperature. Slides were washed again in PBS 3 times and developed using the AEC (3-amino-9-ethylcarbazole) substrate system (Maxim, Fujian, China). Counterstaining was done with hematoxylin. Identical concentration of normal rabbit sera were used as negative control. Sections were examined with an Olympus BX51 microscope and acquired with an Olympus DP12 digital camera controlled by CellSens Standards software.

### Enzyme-linked immunoassay

Mouse sera were collected from mice before and 2, 4, 6, 8, 10 weeks after *S. japonicum* infection. Sera were kept at −80°C until use. Rabbit sera before and after *S. japonicum* infection were kindly provided by Dr. Wei Hu.

Antibody reactivity of animal sera against recombinant prokaryotic SjE16.7 (GST-tag free), eukaryotic His-SjE16.7 and SEA were determined by enzyme-linked immunosorbent assay (ELISA) using adaptations of previously described methods [13]. In short, microtitration plates (Nunc, Denmark, 96 wells, flat bottom) were coated with 100 µl 5 µg/ml SEA, SjE16.7 or His-SjE16.7 respectively. Mouse antisera were diluted 1∶100 and HRP conjugated goat anti-mouse/rabbit IgG or IgM Abs (Sigma) was used as the secondary antibody at a dilution of 1∶1000. Reactions were developed using 3,3′,5,5′-Tetramethylbenzidine (TMB) substrates and stopped with 2 N H_2_SO_4_. The optical densities were read at 450 nm in a microwell reader system (μQuant, Bio-Rad, USA).

### Air pouch model

Dorsal air pouches were induced in mice using previously described methods [14]. In brief, 4 ml of sterile-filtered air was injected subcutaneously into the back of C57BL6/J mice, and the pouch was reinflated with 3 ml of sterile air 3 d later. The dorsal air pouches of groups of 5–6 mice were either injected with 1 ml 0.5% carboxymethylcellulose (CMC) or 0.5% CMC with 50 µg recombinant antigens (His-SjE16.7, GST, or GST-SjE16.7) 3 d later. Four hours or 24 h later, the mice were sacrificed and air pouches were lavaged with 3 ml sterile PBS. The aspirate was centrifuged at 500 g 10 min at 4°C. Supernatants were separated and stored at −80°C until testing. Cell pellets were re-suspended in PBS and counted in a standard hemocytometer chamber.

### Flow cytometry

Cells were incubated with FITC-conjugated anti-CD11b monoclonal antibody (MAb), Percy- Cyanine5.5 conjugated anti-Ly6G (Gr-1) MAb and APC conjugated anti-F4/80 MAb. The cells were analyzed on a FACSCalibur flow cytometer (BD Bioscience) equipped with Cell Quest software. Neutrophils were defined as cells that were CD11b^+^Ly6G^high^F4/80^−^ and macrophages as cells that were CD11b^+^Ly6G^−^F4/80^+^.

### Myeloperoxidase assay

Leukocyte myeloperoxidase (MPO) activity was assessed by measuring the H_2_O_2_-dependent oxidation of TMB as previously reported [15]. Supernatants collected from air pouch aspirates were used for the assay. Aliquots of 30 µl were incubated with 120 µl of TMB substrates in 96 well plates. Plates were incubated for 5 min at room temperature and stopped with 2 N H_2_SO_4_. The optical densities were read at 450 nm in a microwell reader system (μQuant, Bio-Rad, USA). All the samples were performed in duplicates, and samples from the one experiment were tested on the same plate.

### Preparation of peritoneal neutrophils

To prepare peritoneal neutrophils, 6–7 week old mice were injected intraperitoneally with 2% casein/PBS. After 4 hour, peritoneal exudates cells were collected, spun down, and suspended in HBSS supplemented with 0.1% BSA (HBSS/0.1%BSA). Neutrophil viability was >95% according to the results from trypan blue staining. Purity was typically 90% as assessed by flow cytometry based on the forward and side scatter and high Ly6G staining.

### Transwell migration assay

Assay were performed in modified Boyden Chambers (Transwell from Costar, Corning Life Sciences) (6.5 nm in diameter; 3 µm pore size), according to the manufacturer's protocol. In brief, the bottom sides of the inserts were coated with 2 µg/ml fibronectin. Murine neutrophils were plated (1×10^6^/well in DMEM, 0.1% BSA) in the top chamber of Transwell inserts, and 0.1%BSA/DMEM with antigens (SjE16.7 or SEA) was added to the bottom chamber. After incubation for 3 h at 37°C and 5% CO_2_ humidified environment, 50 µl 70 mM EDTA was then added to the top and bottom chambers to release the cells that have adhered to the well and bottom of the membrane. After incubation for 5 min, the number of transmigrated cells in the lower compartment was determined with a hemocytometer.

### PBD pull-down assay

Murine neutrophils (5×10^6^) were treated with 1 µM SjE16.7 or control protein for 10 min or the time as indicated. After treatment, cells were washed in PBS, and lysed in PBD lysis buffer (50 mM Tris pH 7.5, 10 mM MgCl_2_, 0.2 M NaCl, 0.5% NP-40, and 1× protease inhibitors cocktail (Roche)). The lysate was incubated with 20 µg of PAK-GST protein beads (Cytoskeleton) for 30 min at 4°C. After washing, protein on beads and in total cell lysates was subjected to Western blot (Rabbit anti-Rac1/2/3 Ab, Cell Signaling) to determine the level of active Rac.

### Preparation of antigen coupled beads

Glutathione Sepharose 4B beads (GE Healthcare Life Sciences) were incubated with GST or GST-SjE16.7 protein (2 mg antigen/ml beads) for 2 h at room temperature. The mixture was centrifuged for 2 min at 5,000 g, and the resulting pellet was washed twice with 10 bed volumes of sterile PBS. The beads were resuspended in sterile PBS at the concentration of 1×10^5^ beads/ml before injection.

### Induction and evaluation of inflammatory hepatic granulomas in mice

Inflammatory hepatic granulomas were induced in mice by injecting antigen-coupled beads in the cecal vein of the mouse as described [16], [17]. The mouse was anesthetized (60 mg/kg pentobarbital intraperitoneally), and a midabdominal incision was made. 20,000 beads coupled with GST or GST-SjE16.7 dissolved in 0.2 ml of sterile PBS were injected in the cecal vein using an insulin syringe (Becton Dickinson). Unloaded beads were injected as negative control. 4–6 mice were used for each group. To check the role of SjE16.7 in initiation of inflammatory hepatic granulomas, the animals were sacrificed 6 h after injection.

To evaluate the acute granulomatous response, consecutive 3 µm-thick formalin-fixed paraffin-embedded liver sections were stained with H&E. H&E-stained sections were examined with an Olympus BX51 microscope and acquired with an Olympus DP12 digital camera controlled by CellSens Standards software. A quantitative analysis of inflammatory response around the beads (diameter between 50 and 100 µM) was performed by counting the leukocytes in the granuloma. 30 granulomas per mouse were calculated.

### Statistical analyses

Groups were compared using the two-tailed student t test and analysis of variance (ANOVA) with GraphPad Prism 5 software. A nonparametric Mann-Whitney *U*-test was used for analysis of the western blot data because of the relatively small sample size in each experiment. Results were considered significant at a P value of <0.05.

### GenBank accession numbers

The GenBank accession numbers for SjE16.7 and SmE16 are AAW27865.1 and AAA29859.1 respectively. The GenBank accession numbers for *Clonorchis sinensis* calcium-binding protein, *S. mansoni* calmodulin, *C. sinensis* 16 kDa calcium-binding protein and *Fasciola hepatica* CaM3 are GAA27855.1, XP_002580524.1, GAA40982.1 and AFM84631.1 respectively. The GenBank accession number for *S. japonicum* protein SJCHGC05185 is AAW26060.1.

## Results

### Cloning and expression of SjE16.7

**Figure 1:**
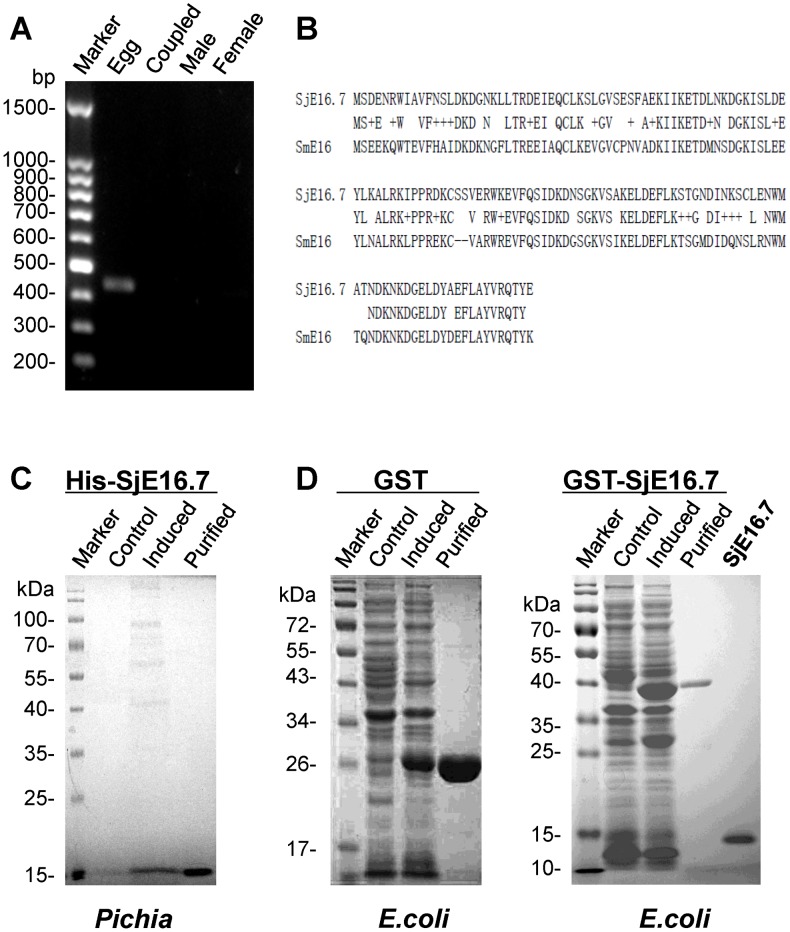
Cloning and expression of SjE16.7. (**A**) RT-PCR with total RNA obtained from female, male, coupled adults and eggs of *S. japonicum*. Equal amounts of total RNA were used for reverse transcription. PCR amplifications were performed with equal amounts of cDNAs and SjE16.7-specific primers. PCR products were separated on an agarose gel. (**B**) Amino acid sequence comparison between SjE16.7 and SmE16 (GenBank AAW27865.1 vs. AAA29859.1). (**C**) SDS-PAGE analysis of recombinant His-SjE16.7 expressed in *P. pastoris* strain GS115. 20 µl Culture supernatant or 2 µg purified His-SjE16.7 were applied to a SDS-PAGE and then stained with Coomassie brilliant blue. Control, GS115/pPIC9K-His-SjE16.7 before induction; Induced, GS115/pPIC9K-His-SjE16.7 after methanol induction. Purified, His-SjE16.7 purified by Ni-NTA column. (**D**) SDS-PAGE analysis of GST or GST-SjE16.7 expressed in *E. coli* BL21. Control, total extract from pGEX-4T or pGEX-4T-SjE16.7 before IPTG induction; Induced, total extract from pGEX-4T or pGEX-4T-SjE16.7 after IPTG induction; Purified, GST or GST-SjE16.7 purified by Glutathione Sepharose 4B; SjE16.7, purified SjE16.7 from *E. coli* after thrombin treatment.

The cDNA clone encoding the full-length sequence of SjE16.7 of *S. japonicum* was obtained by RT-PCR amplification with total RNA extracted from egg. In agree with the results reported by other groups [10], no SjE16.7 cDNA could be amplified from male, female or coupled-adult RNA (**Fig. 1A**). The amplified full-length cDNA sequence of SjE16.7 was verified by sequencing (Majorbio, China). The sequence was comprised a 435 bp ORF encoding 145 amino acid residues with the predicted molecular mass of ∼16.725 kDa and theoretical isoelectric point of pH 4.88. (The molecular mass and isoeletric point of SjE16.7 were calculated using the Compute pI/Mw tool (<http://web.expasy.org/compute_pi/>). Comparison of the predicted amino acid sequence showed that SjE16.7 was 70% identical with the homolog from *S. mansoni* (SmE16) according to multiple sequence alignment analysis (GenBank AAW27865.1 vs. AAA29859.1) (**Fig. 1B**).

To prepare the recombinant SjE16.7 protein, the gene was cloned into eukaryotic expression vector pPIC9K and prokaryotic vector pGEX-4T-1 respectively. The eukaryotic recombinant protein was expressed in the yeast *Pichia pastoris* as His fusion protein (His-SjE16.7) with an expected molecular mass of ∼17 kDa (**Fig. 1C**). Prokaryotic protein was expressed in *Escherichia coli* as a GST-tagged protein (GST-SjE16.7) of ∼42 kDa in size. The molecular weight of purified SjE16.7 after thrombin treatment is around 16 kDa (**Fig. 1D**). After 6 cycles of Triton X-114 phase separation, LPS in GST and GST-SjE16.7 was between 0.10 and 0.15 EU/ml, which is the FDA endotoxin limit for drugs (data not shown).

### Localization and antigenicity of SjE16.7

**Figure 2:**
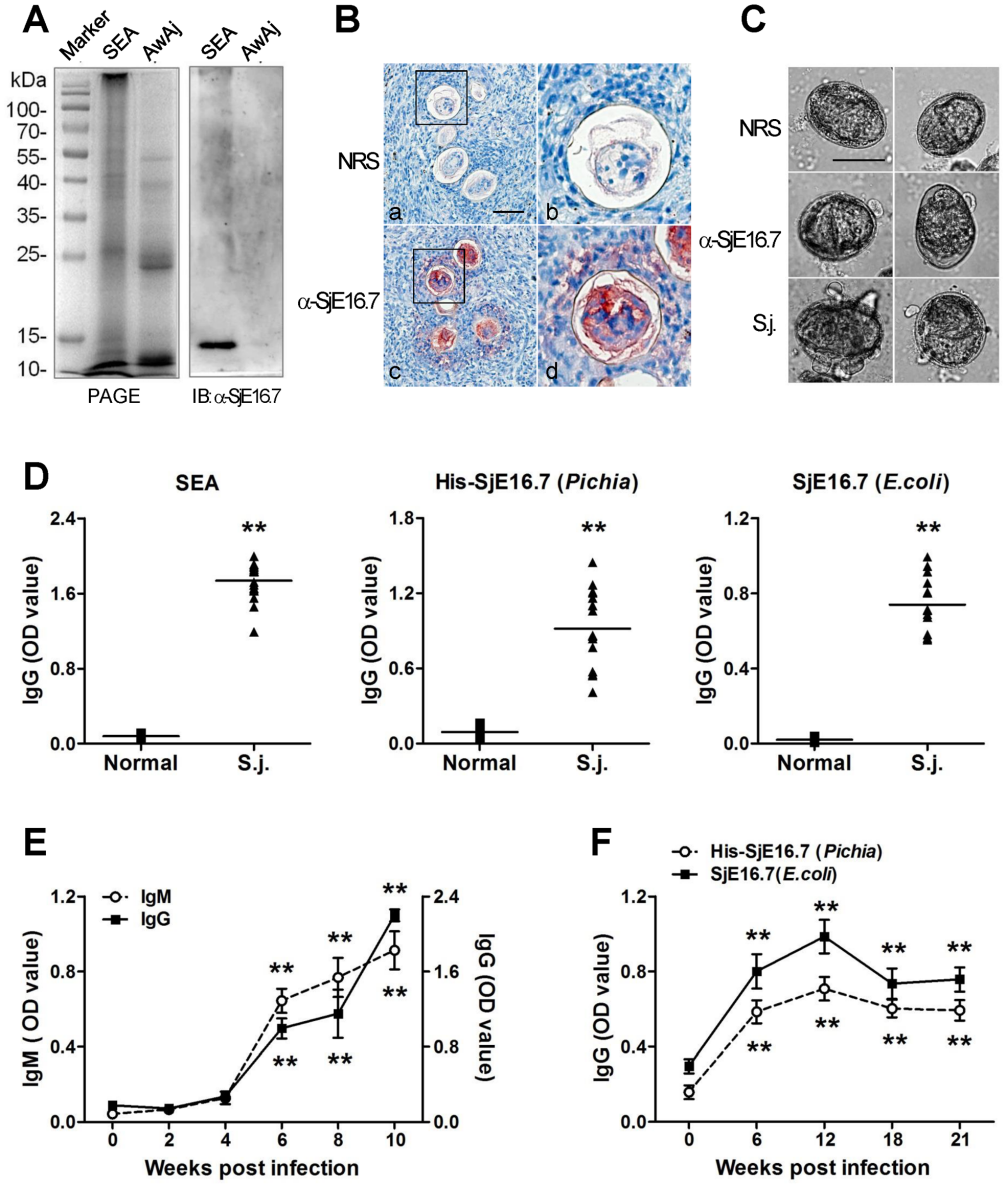
Localization and immunogenicity of SjE16.7. (**A**) Expression of SjE16.7 in the egg stage of *S. japonicum*. Extracts obtained from adult and egg stages of *S. japonicum* were separated by SDS-PAGE and blotted with anti-SjE16.7 sera. SEA, soluble egg antigen. AwAj, adult worm antigen of *S. japonicum*. (**B**) Immunohistological detection of SjE16.7 in *S. japonicum* eggs. Sections of livers from *S. japonicum*-infected mice were incubated with normal rabbit sera (**a,b**) or rabbit anti-SjE16.7 sera (**c, d**) NRS, normal rabbit sera; α-SjE16.7, rabbit anti-SjE16.7 sera. Right graphs are better seen in the magnification of the insets of upper graphs. Original magnification ×40, scale bar = 50 µm. (**C**) Precipitate formation around live eggs cultured in vitro with anti-SjE16.7 sera (α-SjE16.7) and *S. japonicum* infected rabbit sera (S.j.), but not normal rabbit sera (NRS). Original magnification ×40, scale bar = 50 µm. (**D**) ELISA detection of anit-SEA or SjE16.7 IgG in *S. japonicum* infected mice. The sera were collected before (Normal) and 42 days after *S. japonicum* infection (S.j.). SEA, soluble egg antigen; His-SjE16.7, recombinant His-SjE16.7 protein from *Pichia* yeast; SjE16.7, recombinant SjE16.7 (without GST tag) protein from *E. coli*. ** P<0.01. (**E, F**) Time course determined by ELISA. Sera from *S. japonicum* infected mice (E) or rabbits (F) were collected at different time points. Anti-His-SjE16.7 IgG or IgM antibodies were measured by ELISA. Each group consisted of at least 5 animals. Data are presented as optical density at 450 nm (mean ± SD). ** P<0.01 vs. zero time point.

SjE16.7 transcript is highly expressed in egg stage but not in adults. To investigate whether also SjE16.7 protein occurs specifically in the egg stage, a polyclonal mouse antiserum was raised against His-tagged SjE16.7. We analyzed protein extracts obtained from adult and egg stages of SjE16.7 by SDS-PAGE followed by Western blotting. To ensure that comparable amount of proteins of each stage were present, the samples were duplicated. One SDS-PAGE gel was stained with Coomassie brilliant Blue. The other gel was transferred to the membrane and then blotted with mouse SjE16.7 antiserum. As on the mRNA level, SjE16.7 could be clearly detected only in the egg stage (**Fig. 2A**). Concerning the site of expression of SjE16.7 within the eggs, sections of *S. japonicum*-infected mouse liver were stained with rabbit anti-SjE16.7 serum. Immunohistochemistry results showed that inside the egg, SjE16.7 locates in the miracidium and presents between the eggshell and miracidium, the subshell area of the egg (**Fig. 2B**). SjE16.7 also shows within the granuloma surrounding the egg which indicates it is secreted by the egg. To further clarify that SjE16.7 is a secretion protein, we performed circumoval precipitin tests (COPT). Live schistosome eggs were cultured at 37°C with normal rabbit sera or anti-SjE16.7 sera *in vitro* for 48 hours. The formation of precipitates showed around the eggs cultured with anti-SjE16.7 sera but not eggs cultured with normal sera (**Fig. 2C**)

As an egg specific protein, SjE16.7 can be secreted by the egg. We then asked the antigenicity of SjE16.7, whether this protein provokes immune responses during infection with schistosomes. To address this question, mice were infected with *S. japonicum* cercariae. The antibody production against complete SEA, or SjE16.7 (prokaryotic SjE16.7 or eukaryotic His-SjE16.7) was detected by ELISA respectively. The ELISA results showed that the infected mice developed specific antibodies against SjE16.7 as SEA, while sera from control mice without infection revealed no specific antibodies (**Fig. 2D**). The levels of specific IgM and IgG antibodies were increased significantly 6 weeks after infection and remained at high level until the mice were sacrificed at 10 weeks after infection (**Fig. 2E**). Similar phenotypes were observed in *S. japonicum* infected rabbits. As shown in **Fig. 2F**, 6 weeks post infection, the infected rabbits develop significantly enhanced antibodies against SjE16.7. The antibody levels reach a peak 12 weeks post infection. Although the antibody levels declined in the late infection, compare to the antibody levels before infection, the differences are still significantly.

### SjE16.7 induces inflammatory cell infiltration in murine air pouch model

**Figure 3:**
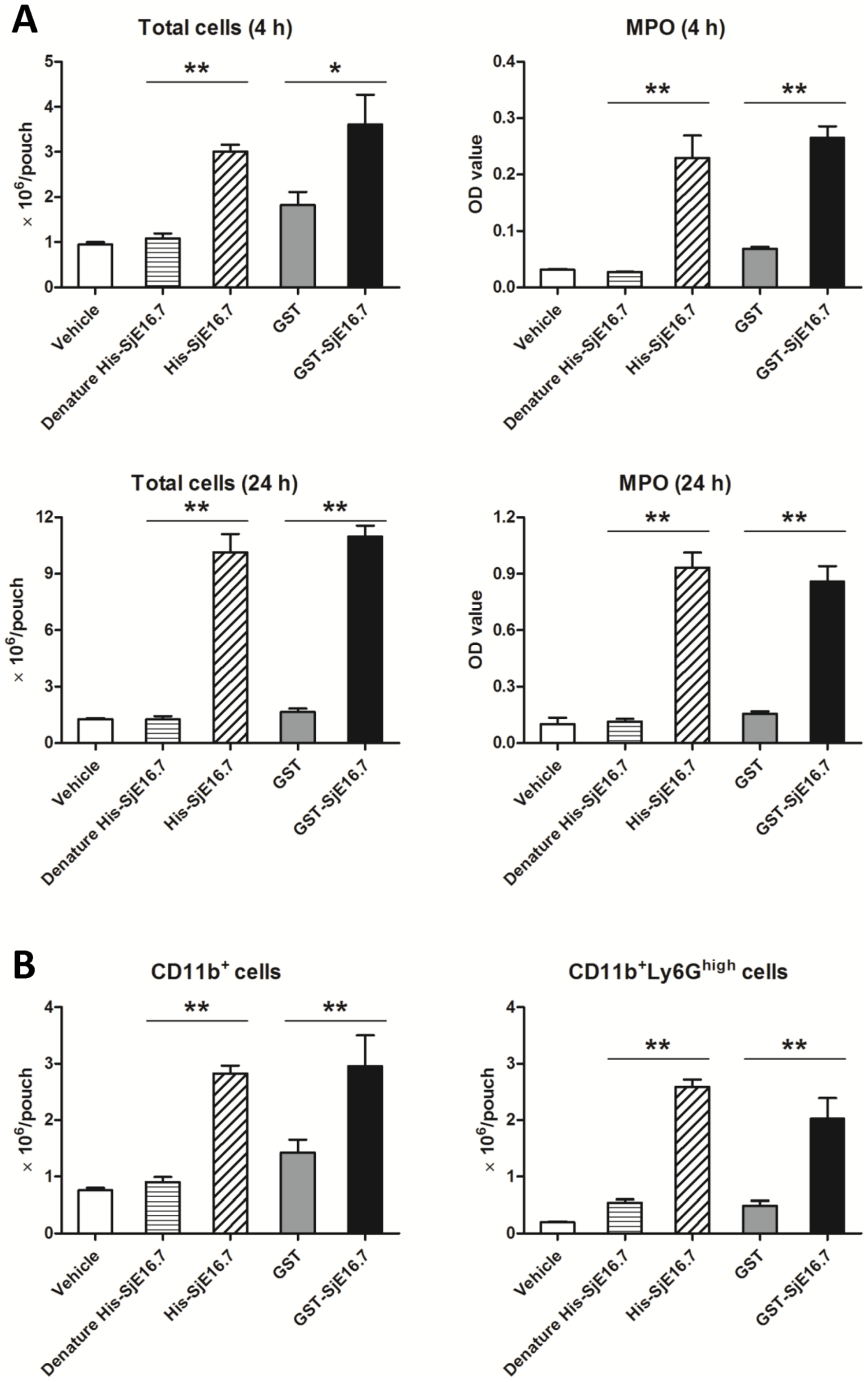
SjE16.7 induces inflammatory cell infiltration in murine air pouch model. (**A**) Total leukocyte number and myeloperoxidase (MPO) level in the exudates of mice with 6-day-old air pouches. Mice were treated with CMC vehicle or recombinant protein in the vehicle. Equal amounts of denature His-SjE16.7 (95°C, 30 min treatment) or GST were used as control. (**B**) Flow cytometry analysis of cell population in the exudates of mice (4 h after antigen injection). Values are means ± S.E.M (n = 6–7 mice per group; from two independent experiments). *P<0.05, **P<0.01.

Schistosome eggs are highly immunogenic. Within infected hosts, eggs induce vigorous immune responses and are rapidly surrounded by inflammatory cells, creating a granuloma. Above results suggest that SjE16.7 is an egg specific antigen and recognized early during infection with *S. japonicum* in mice. To investigate the role of SjE16.7 in induction of inflammation, we used a murine air pouch model of inflammation. As shown in **Fig. 3**, injection of vehicle or control protein produced a modest infiltration of the cells into the pouch. In contrast, administration of either eukaryotic or prokaryotic recombinant SjE16.7 produced an intense accumulation of inflammatory cells at 4 h and 24 h. Myeloperoxidase (MPO) is one of the principal enzymes released from neutrophils during inflammatory responses. Consistent with enhanced inflammatory cells, the level of MPO was found increased significantly in SjE16.7 induced exudates (**Fig. 3A**). The analysis of cell population in the exudates induced by SjE16.7 showed CD11b^+^ myeloid cells, especially CD11b^+^Ly6G^high^ neutrophils were the predominant cell type (**Fig. 3B**).

### SjE16.7 promotes neutrophil chemotaxis by activating Rac GTPase pathway

**Figure 4:**
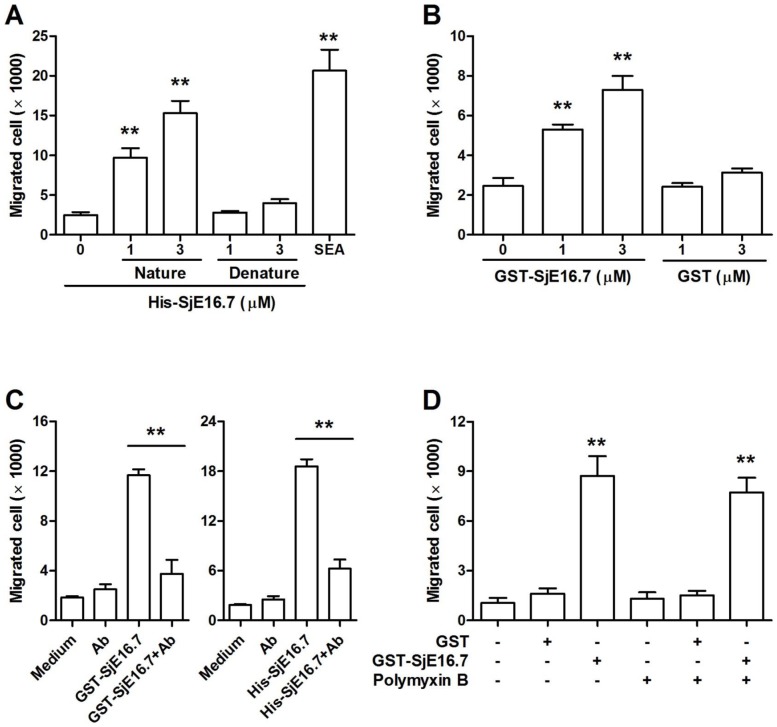
SjE16.7 induces neutrophil migration in vitro. (**A**) Cell migration was measured using transwell assay. Neutrophils were plated in the upper chamber of the filter that had been coated with fibronectin, and stimulated with denature His-SjE16.7 (95°C, 30 min treatment) or His-SjE16.7 as indicated concentration for 3 h. Cells migrating to the bottom chambers were counted. Soluble egg antigen (SEA, 10 µg/ml) was used as control. Values are means ± S.E.M of 4 independent assays. **P<0.01 vs. group without antigen stimulation. (**B**) Transwell assays were performed as described in A, except that cells were stimulated with prokaryotic protein GST or GST-SjE16.7 as indicated. Values are means ± S.E.M of 3 independent assays. **P<0.01 vs. group without antigen stimulation. (**C**) Transwell assays were performed as described in A, except that cells were stimulated with recombinant SjE16.7 with or without purified anti-SjE16.7 antibody (10 µg/ml) as indicated. Values are means ± S.E.M of 2 independent assays. **P<0.01. (**D**) Transwell assays were performed as described in B, cells were stimulated with recombinant GST or GST-SjE16.7 with or without Polymyxin B (10 µg/ml) as indicated. Values are means ± S.E.M of 2 independent assays. **P<0.01 vs. group without antigen stimulation.

SjE16.7 recruited neutrophil infiltration *in vivo*. To further address the effect of SjE16.7 on chemotaxis of neutrophil, we examined migration of the cells in a transwell chamber assay. As shown in **Fig. 4A and 4B**, the presence of SEA or SjE16.7 in the bottom chamber dramatically promoted migration and the function of SjE16.7 was dose dependent. Furthermore, the attraction of neutrophils by SjE16.7 can be neutralized by anti-SjE16.7 antibody (**Fig. 4C**). Prokaryotic SjE16.7 protein was prepared from *E.coli*. We removed majority LPS (from 10 EU/ml to <0.25 EU/ml) in antigen purification, but the residual LPS contamination still might be a chemoattractant for neutrophils. To test this possibility, LPS inhibitor, Polymyxin B was added in our chemotaxis assay. As shown in **Fig. 4D**, Polymyxin B didn't inhibit prokaryotic SjE16.7 induced neutrophil migration.

**Figure 5:**
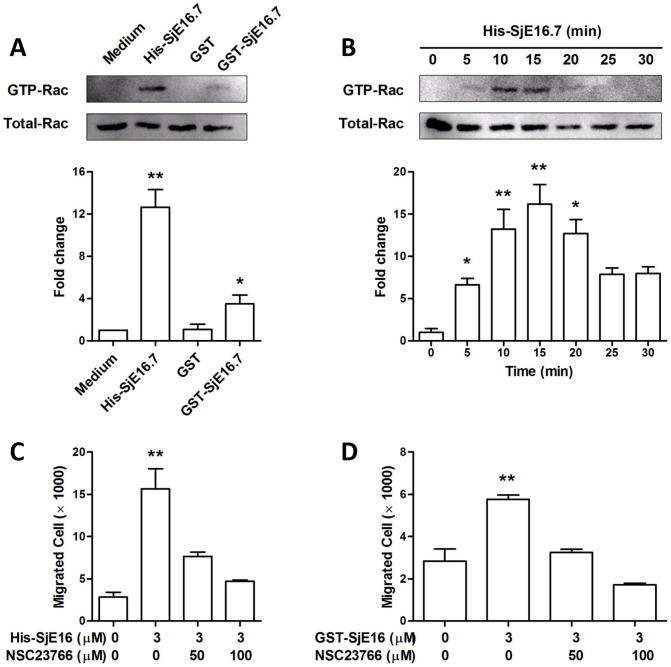
SjE16.7 promotes neutrophil chemotaxis by activating Rac GTPase pathway. (**A**) Murine neutrophils were treated with 1 µM His-SjE16.7, GST or GST-SjE16.7 for 10 min. Cell lysates were subjected to pull-down, using PAK-GST protein beads. Rac from the pull-down and total Rac in the lysates were detected by Western blot. Bar graphs show relative GST-Rac levels as determined by densitometry. Statistics were performed on pooled data from three independent experiments. (**B**) Neutrophils were treated with 1 µM His-SjE16.7 as indicated time. Active and total Rac levels in the cells were determined as in A. (**C**) Neutrophils were pretreated with Rac1 inhibitor NSC23766 at the indicated concentration for 30 min. Cell migration was then measured using transwell assay. Pretreated cells were plated in the upper chamber of the filter that had been coated with fibronectin, and stimulated with 3 µM His-SjE16.7 for 3 h. Cells migrating to the bottom chambers were counted. (**D**) Inhibition assays were performed as described in C, except that cells were stimulated with prokaryotic GST-SjE16.7. Statistics were performed on pooled data from three independent experiments. Values are means ± S.E.M of 3–4 independent assays. *P<0.05, **P<0.01 vs. medium group or group without treatment.

Since cell movement requires dynamic reorganization of the action cytoskeleton and membrane polarization, and both of them regulated by Rac GTPases, we measured Rac GTPase activation in SjE16.7 treated neutrophils. Using GST pull-down assay that specifically recognized active GTP-bound Rac, we tested the SjE16.7 effect on neutrophils. We found that SjE16.7 treated neutrophils exhibited an increase in active Rac levels over controls (**Fig. 5A**). SjE16.7 treatment induces an intense accumulation of GTP-Rac that peaked at 10–20 min, declining over 25 min (**Fig. 5B**). To determine whether SjE16.7 regulates chemotaxis through Rac, in transwell assay neutrophils were pretreated with Rac antagonist NSC23766. As shown in **Fig. 5C** and **Fig. 5D**, NSC23766 depressed the SjE16.7 induced cell chemotaxis in a dose-dependent manner.

### SjE16.7 induces inflammatory hepatic granuloma initiation *in vivo*

**Figure 6:**
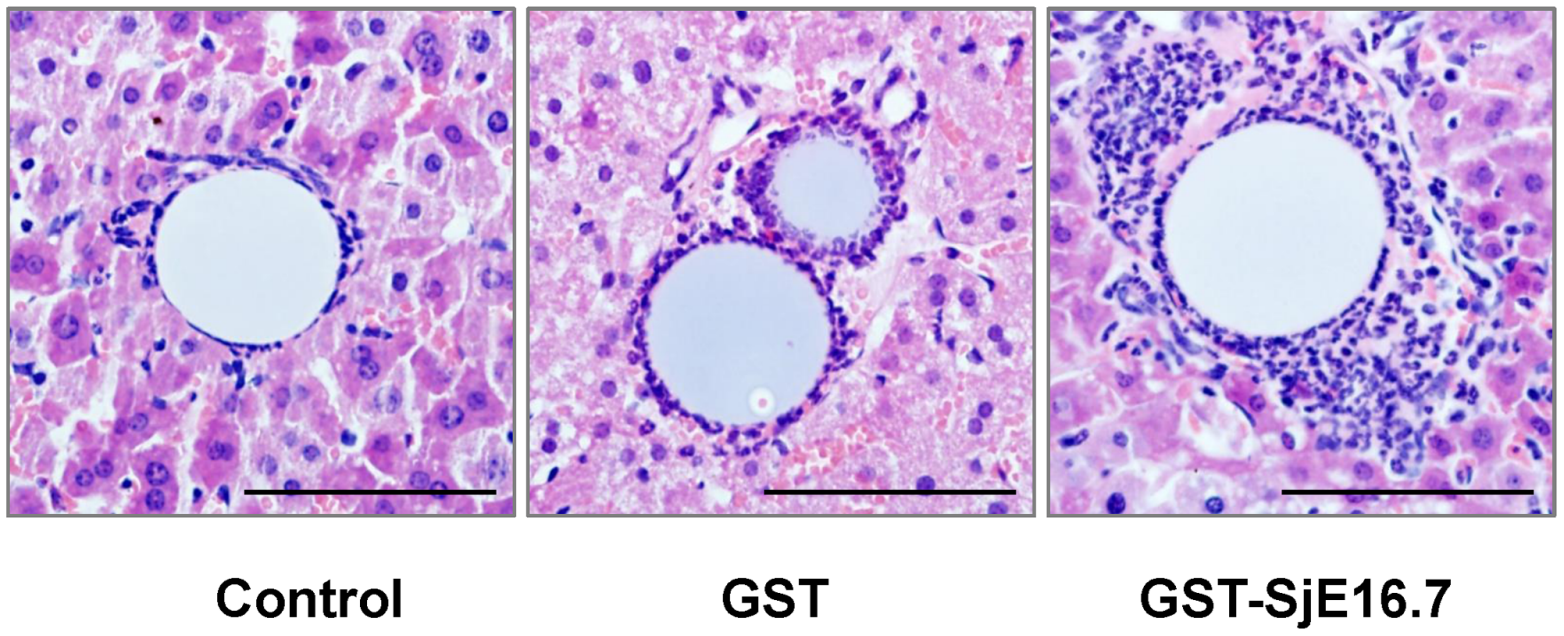
SjE16 induces inflammatory hepatic granuloma in vivo. Antigen (GST or GST-SjE16.7) coupled or uncoupled beads (Control) were injected into C57BL6/J mouse liver as described in material and methods. The animals were sacrificed 6 h after injection and inflammatory hepatic granulomas were analyzed by histochemistry (H & E stain, scale bar = 100 µm). A quantitative analysis of inflammatory response around the beads (diameter between 50 and 100 µm) was performed by counting the leukocytes in the granuloma. 30 granulomas per mouse were calculated. GST-SjE16.7 beads recruited more leucocytes (78.88±15.83/bead) than control beads (12.78±13.31/bead) or GST beads (39.64±10.73/bead). The difference between GST-SjE16.7 group and GST group or control group was significant (GST-SjE16.7 vs. GST, P<0.01; GST-SjE16.7 vs. control, P<0.001).

In schistosome infection, neutrophils are known as a major cellular component in the early phase of egg-associated granulomatous lesion and they are important in initiation of egg granuloma. We next measured the ability of SjE16.7 in initiation of mouse hepatic granuloma. Agarose beads were coated with GST-SjE16.7 or GST control protein. C57BL6/J mice were injected with 20,000 beads and sacrificed at 6 h after injection. Beads were found lodged in the peripheral perisinusoidal ramifications of the portal vein. Uncoated beads and beads coated with control protein showed no cellular reaction, except for a monolayer of leucocytes (**Fig. 6**). Beads coated with GST-SjE16.7 elicited significant granulomatous inflammatory reaction. Quantification of the leucocytes around the beads revealed that GST-SjE16.7 beads recruited more leucocytes (78.88±15.83/bead) than control beads (12.78±13.31/bead) or GST beads (39.64±10.73/bead). The difference between GST-SjE16.7 group and GST group or control group was significant (GST-SjE16.7 vs. GST, p<0.01; GST-SjE16.7 vs. Control, p<0.001).

## Discussion

Neutrophils are known to play a major role in the disease caused by *S. japonicum* [4]. For example, in the initiation of egg-induced granulomatous pathology, neutrophils are recruited to the egg and become the dominant cell population. The accumulating neutrophils cause necrotic lesions in the liver or intestinal tissue of the host. In this study, we identified SjE16.7 as a potent neutrophil recruiter derived from *S. japonicum* egg. It significantly induces neutrophil chemotaxis via Rac GTPase pathway and stimulates neutrophil infiltration *in vivo*. We propose SjE16.7 is an important pathogenic factor which facilitates eggs to migrate through gut tissues and also initiate pathology in the liver. It is a potential target for the disease prevention and treatment.

SjE16.7 transcript was firstly identified by Hu and her colleagues. They compared the expressed sequence tags (ESTs) derived from cercariae, schistosomulae, adults and eggs of *S. japonicum*, then found transcript encoding SjE16.7 is expressed specifically in egg stage [10]. Consistent with this result, our PCR results revealed that SjE16.7 mRNA is restricted to the egg stage. Furthermore, Western blotting performed on the adult and egg stages of *S. japonicum* confirmed that at the protein level, SjE16.7 is present in eggs, and not detectable in adult worms.

Searching with BLASTP at the NCBI database, SjE16.7 displays besides 70% identity with *S. mansoni* SmE16, 43% identity with *S. mansoni* calmodulin, 41% and 39% identity with *Clonorchis sinensis* calcium-binding protein and 16 kDa calcium-binding protein respectively, and 38% identity with *Fasciola hepatica* CaM3. SmE16 is most related with SjE16.7, while the *S. mansoni* calmodulin, 16 kDa calcium-binding protein of *C. sinensis* and *F. hepatica* CaM3 are more related to another *S. japonicum* protein SJCHGC05185. SmE16 is an *S. mansoni* egg specific calcium-binding protein, firstly reported by Moser in 1992 [18]. Mathieson and Wilson compared the proteome of the undeveloped and developed *S. mansoni* egg and showed SmE16 was expressed at a low level in undeveloped, but increased in abundance in developed eggs [19]. In mature eggs, SmE16 could be assigned to miracidium and the water-soluble protein washed from the egg as the miracidium emerged (hatch fluid). These results are consistent with SjE16.7 distribution in the egg. SjE16.7 presents in the miracidium and subshell area of the *S. japonicum* egg. To identify egg secretin proteins of *S. mansoni*, Mathieson and Wilson collected egg secretion protein by incubating mature eggs in serum-free RPMI. In their study egg secretion protein was characterized by multiple isoforms/variants of just 6 proteins and SmE16 was not in it. However in the similar work done by another group in the Unite State, SmE16 was identified as one of the abundant *S. mansoni* egg secreted proteins [20].

As an egg specific protein, SjE16.7 is a secretion protein and can be recognized by the host immune system. This conclusion is based on the following observations: (a) In COPT, the formation of precipitates showed around the live eggs cultured with anti-SjE16.7 sera *in vitro*. (b) In immunocytochemistry, SjE16.7 was detected within the egg and within the granuloma surrounding intact eggs in the liver of mice with *S. japonicum* infection. (c) 6 weeks post *S. japonicum* infection, animals develop significantly specific antibodies against SjE16.7. Immunohistochemstry staining of *S. japonicum* eggs in liver sections of infected mice revealed that SjE16.7 locates in the miracidium and subshell area of the egg. This subshell area is supposed to be responsible for egg secretion protein synthesis and storage [21]. Several secretary proteins, such as IPSE/alpha-1, Omega-1 and Kappa-5 have been found in this area [22]–[24]. SjE16.7 shows in subshell area, but whether it is synthesized in that area, or it is a secretary/excretory protein produced by miracidium and stored in subshell area is presently under study.

The localization and its antigenic properties strongly suggest a role for SjE16 as an egg-derived molecule involved in host-parasite interactions. Analysis of SjE16.7 protein sequence revealed SjE16.7 has 2 EF-hand domains. The classical EF-hand is composed of 2 perpendicular alpha helices separated by a loop, which form a helix-loop-helix motif. The loop integrated in this motif can accommodate Ca^2+^ or Mg^2+^ with distinct geometries and the affinity for these ions is determining factor for the function of the protein [25]. Previous studies of schistosome showed proteins containing EF-hand domain may play roles in modulating host immune responses during the process of host-parasite interaction [26]. Sm22.6 and Sm20.8 are schistosome tegumental antigens exclusively expressed in adult worms. Both of them have 2 EF-hand motifs and are dominant targets for human IgE responses in the process of schistosome infection and therefore, are important for parasites survival [27], [28]. SmE16 is a stage specific calcium-bind protein expressed in eggs of *S. mansoni*. It has two EF-hands and share 70% identical sequence with SjE16.7. The detection of specific antibodies to the SmE16 in sera of schistosomiasis patients suggests a role for SmE16 as an egg-derived molecule involved in host-parasite interactions, but the function of this protein in the egg is still unclear [18].

EF-hand structural motif has been found in a large number of protein families, possessing diverse functions. In inflammation, EF-hand proteins participate in many steps of inflammatory processes, including leukocyte migration, inflammatory cell activation, cytokine production and et al. [25]. For example, mammalian S100A8 and S100A9 are members of S100 family which has 2 EF-hand motifs. They are regarded as marker proteins for a number of inflammatory diseases [29]. Studies in human and mouse showed S100A8 and S100A9 have potent chemotactic activity for neutrophils and monocytes [29], [30]. They regulate leukocytes trafficking by acting as a chemoattractant and influencing expression of adhesion molecules. Similar to S100A8 and S100A9, SjE16.7 has 2 EF-hand motifs and potent chemotactic activity for neutrophils. It might be an EF-hand protein used by parasite to mediate the inflammatory responses which facilitate egg passage through the tissues.

Neutrophils are highly motile leukocytes. For neutrophil migration to occur there is a constant need for the cell to coordinate a variety of intracellular activities both spatially and temporally [31]. The small Rho guanosine triphosphatases (GTPases) Rac1 and Rac2 are key players in this process and their ability to cycle between active (GTP-bound) and inactive (GDP-bound) status allows the cell to respond rapidly to extracellular signals. Regulating membrane polarization or cytoskeletal dynamics, Rac GTPases promote cell migration. The up-regulation of GTP-Rac in SjE16.7 treated neutrophils suggested SjE16.7 may induce cell migration trough Rac. This mechanism was confirmed by Rac antagonist NSC23766 which depressed the SjE16.7 induced cell migration. The major activator of Rho family GTPase is G-Protein coupled Receptor (GPCR) [32]. It has been reported GPCR was used by other EF-hand protein to stimulate myeloid cell chemotaxis [33]. Whether SjE16.7 uses the same receptor to activate Rac and simulate neutrophil migration remains to be determined. Besides Rac GTPases, a huge variety of intracellular signaling molecules have been implicated in neutrophil migration, including MAPK cascades, PI3Ks, phospholipases, scaffold proteins and other GTPases [31], [34]. In the cells, those signaling molecules interplay and cooperate with each other, and then cause the cell migration. For example, after receptor-chemoattractant interaction, PI3Ks activation leads to PIP3 accumulation at the leading edge of the cells. PIP3 coordinates Rac-GEFs recruitment and in turn Rac activation that allows actin polymerization. In this study, we checked Rac GTPase in SjE16.7 induced neutrophil chemotaxis, however other signaling molecules in neutrophil migration are still worth to be considered.

The granulomatous response to the egg is a dynamic process. It is orchestrated by neutrophils, macrophages, CD4^+^ T cell, and multiple other cell types. In this study we focus on the relationship between SjE16.7 and neutrophil, and found it recruits neutrophil infiltration. It would be interesting to know the effects of SjE16.7 on other cell types and whether it influences on the development and resolve of granuloma. Macrophages are one of the major cells populations in egg granuloma. They are involved in the initiation of granuloma formation [35] and play essential roles in granuloma developing and resolving. In our hands, SjE16.7 dramatically chemoattracts macrophages as neutrophils (our unpublished data). Moreover, SjE16.7 enhanced the cytokine expression and secretion in macrophages through intracellular MAPK signal pathway (our unpublished data). Macrophage recruitment and activation are essential for the development and resolve of the granuloma. For example, the presence of alternative active macrophages (AAMs) in the granuloma downmodulates the initiate inflammatory responses and provides a readily available supply of proline to the fibroblasts resulting in collagen synthesis [2]. SjE16.7 promotes macrophage migration may contributes to the initiation of the granuloma. Whether SjE16.7 induced macrophage activation is associated with AAM polarization, therefore affects granuloma development is worth to be studied. An effective T-cell response is known to be critical for the development of the granulomatous response. To date, we haven't worked on it yet. Further work is required to know the relationship between SjE16.7 and other cell types, and fully elucidate roles of SjE16 in the developing and resolving granuloma.
